# Is C-type natriuretic peptide regulated by a feedback loop? A study on systemic and local autoregulatory effect

**DOI:** 10.1371/journal.pone.0240023

**Published:** 2020-10-01

**Authors:** Yohei Ueda, Keisho Hirota, Ichiro Yamauchi, Takuro Hakata, Takafumi Yamashita, Toshihito Fujii, Akihiro Yasoda, Nobuya Inagaki

**Affiliations:** 1 Department of Diabetes, Endocrinology and Nutrition, Kyoto University Graduate School of Medicine, Sakyo-ku, Kyoto, Japan; 2 Department of Metabolism and Endocrinology, Kishiwada City Hospital, Kishiwada-shi, Osaka, Japan; 3 Clinical Research Center, National Hospital Organization Kyoto Medical Center, Fukakusa, Fushimi-ku, Kyoto, Japan; Max Delbruck Centrum fur Molekulare Medizin Berlin Buch, GERMANY

## Abstract

C-type natriuretic peptide (CNP) is a pivotal enhancer of endochondral bone growth and is expected to be a therapeutic reagent for impaired skeletal growth. Although we showed that CNP stimulates bone growth as a local regulator in the growth plate via the autocrine/paracrine system, CNP is abundantly produced in other various tissues and its blood concentration is reported to correlate positively with growth velocity. Therefore we investigated the systemic regulation of CNP levels using rodent models. In order to examine whether CNP undergoes systemic feedback regulation, we investigated blood CNP levels and local CNP expression in various tissues, including cartilage, of 4-week-old rats after systemic administration of sufficient amounts of exogenous CNP (0.5 mg/kg/day) for 3 days. This CNP administration did not alter blood NT-proCNP levels in male rats but decreased mRNA expression only in tissue that included cartilage. Decrease in expression and blood NT-proCNP were greater in female rats. To analyze the existence of direct autoregulation of CNP in the periphery as an autocrine/paracrine system, we estimated the effect of exogenous supplementation of CNP on the expression of endogenous CNP itself in the growth plate cartilage of extracted fetal murine tibias and in ATDC5, a chondrogenic cell line. We found no alteration of endogenous CNP expression after incubation with adequate concentrations of exogenous CNP for 4 and 24 hours, which were chosen to observe primary and later transcriptional effects, respectively. These results indicate that CNP is not directly autoregulated but indirectly autoregulated in cartilage tissue. A feedback system is crucial for homeostatic regulation and further studies are needed to elucidate the regulatory system of CNP production and function.

## Introduction

C-type natriuretic peptide (CNP) is the third member of the natriuretic peptide family along with atrial natriuretic peptide (ANP) and brain natriuretic peptide (BNP) [1, 2]. ProCNP, a progenitor of CNP, is produced in various tissues such as the brain, blood vessels, cartilage, and bone [3–9]. ProCNP is cleaved by furin into amino-terminal proCNP (NT-proCNP) and CNP-53 and CNP-53 is cleaved again to yield CNP-22 [10]. CNP-53 and CNP-22 are bioactive forms of CNP, which increase intracellular cGMP levels by binding to natriuretic peptide receptor B (NPR-B). Past studies using knockout rodents [9, 11, 12] and transgenic mice [13, 14] have revealed that CNP stimulates endochondral ossification and promotes linear growth. In humans several genetic diseases with impaired skeletal growth [15–21] or skeletal overgrowth [22–27] are linked to the CNP/NPR-B system. Thus CNP is a pivotal physiological stimulator of endochondral bone growth in humans and is now greatly anticipated to be a therapeutic reagent for the impaired skeletal growth observed in genetic disorders such as achondroplasia [13, 28, 29] or caused by drugs such as glucocorticoids [30, 31].

Although systemic administration of CNP was an approach that raises plasma CNP concentration, which was thought to be effective in the treatment of impaired skeletal growth, we previously generated and studied cartilage-specific CNP or NPR-B knockout mice and showed that CNP was a local regulator of the growth plate cartilage, promoting skeletal growth through an autocrine/paracrine mechanism rather than an endocrine mechanism [32]. Intracellular CNP/NPR-B activity could be related to circulating CNP level. Patients with homozygous loss of function mutations in *NPR2*, a coding gene for NPR-B, have high blood CNP levels [16, 21]. These alterations of CNP levels suggest the presence of feedback system of CNP. However, blood CNP levels of patients with heterozygous loss-of-function mutations in *NPR2* have been reported to be within the normal range even though they exhibit short stature [16]. As for gain-of-function mutations in *NPR2*, while one clinical case with an NPR-B activating mutation was reported to have low circulating NT-proCNP levels [26], another case was reported not to have low NT-proCNP levels [27]. Regulation of CNP production have been not fully studied.

We think that it is important to comprehend the systemic regulatory system of CNP in case we use it or an analogue as a remedy. In this study, we intended to clarify whether there exists systemic or endocrinological regulation of blood CNP levels, i.e., feedback regulation of CNP, in *in vivo* experiments using wild-type rats. A feedback loop is key to hormonal regulation and analyses to confirm its existence would be very meaningful. Next we examined the effect of CNP on CNP production in various tissues that could affect plasma CNP concentrations. Finally, because the local effect of CNP is supposed to be vitally important in stimulating endochondral bone growth, we analyzed the existence of CNP autoregulation in chondrocytes using growth plates of tibial explants from fetal rats and a chondrogenic cell line, ATDC5.

## Materials and methods

### Animals

All experimental procedures involving animals were approved by the Animal Research Committee, Graduate School of Medicine, Kyoto University (Permit number: MedKyo07598). Care of animals and all animal experiments were conducted in accordance with the institutional guidelines of Kyoto University Graduate School of Medicine. The animals were housed in a humidity and temperature-controlled environment with an automatic 12-hour light/dark cycle. They were fed a standard pelleted lab chow diet (CRF-1; Oriental Yeast Co., Ltd., Japan) and tap water ad libitum. Surgical procedures were performed under isoflurane-induced anesthesia and carbon dioxide was used for euthanasia. All efforts were made to minimize suffering.

Animal experiments on CNP knockout rats and wild-type rats were performed using F344/Stm rats deposited with the National Bio Resource Project for Rats in Japan (www.anim.med.kyoto-u.ac.jp/nbr). CNP knockout rats were generated in the F344/Stm background by the method previously reported [12] and we used the homozygous Δ774 mutant rats described in the report.

CNP transgenic mice under the control of human serum amyloid P component (SAP) promoter (SAP-*Nppc*-Tg mice) were generated in the C57BL/6J background by the method previously reported and we used SAP-*Nppc*-Tg line 17 described in the report for the following experiments. These mice harbor the human SAP / mouse CNP fusion gene and produce excessive CNP in their livers, resulting in higher plasma CNP levels than wild-type mice [14].

We used male rodents for the following experiments unless otherwise noted because CNP mRNA expression is stimulated by GnRH [33] and therefore CNP production in female rats would be variable during the estrus cycle.

### Organ culture

On day 16.5 of pregnancy, rats were sacrificed and humeruses, radiuses, ulnas, femurs, tibias, and fibulas were resected from fetal rats. These bones were cultured for 24 hours in BGJb medium (No.12591-038, Gibco) with 6 mg/ml of albumin from bovine serum (No. 010–23382, Wako), 150 μg/ml of ascorbic acid (No. 012–04802, Wako), and 10 μl/ml of penicillin-streptomycin solution (No. 168–23191, Wako). The tibias were incubated at 37°C in a humidified atmosphere of 5% CO_2_.

### Cell culture

A chondrogenic cell line of ATDC5 cells [34] was purchased from RIKEN CELL BANK (No. RCB0565, RIKEN CELL BANK, Tsukuba, Japan). The cell line was authenticated by RIKEN CELL BANK. Cells were maintained with Dulbecco’s modified Eagle’s Medium/Nutrient Mixture F-12 Ham (No. D6421, SIGMA) containing 5% fetal bovine serum (No. 10270–106, Thermo Fisher Scientific), 100 U/ml penicillin, and 100 μg/ml streptomycin (No. 26253–84, Nacalai) at 37°C in a humidified atmosphere of 5% CO_2_. The medium was replaced every other day.

### Administration of CNP

CNP-53 was purchased from PEPTIDE INSTITUTE, INC. (Ibaraki, Japan) as human CNP-53 (No. 4241-s, PEPTIDE INSTITUTE) and dissolved in water to a concentration of 50 μg/ml.

Rats were treated with 10 ml/kg/day of water as a vehicle or 0.5 mg/kg/day (10 ml/kg/day) of CNP-53. Water and CNP-53 were administered using osmotic pumps (ALZET® osmotic pump 1007D, Durect Corporation, CA, USA). Osmotic pump 1007D is designed to continuously release drugs for 7days. Pumps containing 7 days of vehicle or CNP were surgically implanted subcutaneously. The dose of 0.5 mg/kg/day was determined in accordance with our previous report in which this dose of CNP-53 restored the dwarfism of CNP knockout rats [35].

### Measurement of NT-proCNP

Four-week-old rats were treated with vehicle or CNP-53 in the same manner described above. After 3 days of administration, blood was collected and serum NT-proCNP levels, as the marker of CNP production, were measured using proCNP, N-terminal, EIA Kit (BI-20812, Biomedica Medizinprodukte GmbH & Co KG, Wien, Austria). This kit is designed to measure human NT-proCNP by using a sandwich assay. Rat NT-proCNP shares a 92% homology to human NT-proCNP and the rat serum samples were verified for this kit according to a spike recovery test (97%) and a linearity test (87%). The treatment duration was determined in accordance with our previous report in which 3 days of glucocorticoid treatment suppressed the CNP production in wild-type rats [30]. As a control group, NT-proCNP levels in 4-week-old rats without interventions were also measured.

### Measurement of cGMP

ATDC5 cells were plated at 1.0 × 10^5^ cells/well in 6-well tissue culture plates. ATDC5 cells were differentiated into proliferative chondrocytes by incubation with 10 μg/ml bovine insulin (No. 10516, SIGMA) for 14 days. Differentiated ATDC5 cells were incubated with vehicle or 10^−7^ M CNP-22 for 30 minutes. Culture media were acetylated and their cGMP level were measured using the cGMP ELISA kit (No. 581021, Cayman Chemical, Michigan, USA).

### Quantitative RT-PCR analysis

Four-week-old rats were treated for 3 days as described above, and were then sacrificed and their lumbar vertebrae, blood vessels, cerebrums, and tibial diaphyses were resected. Total RNA was extracted from the tissues using RNeasy Lipid Tissue Mini Kit (No. 74084, QIAGEN). One μg of total RNA was reverse-transcribed using ReverTra Ace (No. TRT‐101, TOYOBO Life Science, Osaka, Japan). Quantitative PCR analysis was performed using THUNDERBIRD SYBR qPCR MIX (No. QPS-201, TOYOBO Life Science) with the StepOnePlus™ Real-time PCR System (Thermo Fisher Scientific, Massachusetts, USA).

For the analysis of cerebrums, results were normalized using rat β-glucuronidase (*Gusb*) and rat 3-monooxygenase/tryptophan 5-monooxygenase activation protein, zeta polypeptide (*Ywhaz*), as reference genes. The reference genes for blood vessels were *Ywhaz* and rat glyceraldehyde-3-phosphate dehydrogenase (*Gapdh*). The reference genes for both lumbar vertebrae and tibial diaphyses were rat hypoxanthine phosphoribosyltransferase 1 (*Hprt1*) and rat peptidylprolyl isomerase A (*Ppia*). These reference genes were selected based on past reports [36–38].

Four-week-old wild-type mice and SAP-*Nppc*-Tg mice were also sacrificed and their lumbar vertebrae were resected. RNA extraction, reverse-transcription, and quantitative PCR analysis were performed as described above. Results were normalized using *Hprt* and *Ppia* as reference genes.

For *ex vivo* experiments, limb bones from rat fetuses were incubated in 12-well tissue culture plates with vehicle or 10^−7^ M CNP-22 for 24 hours. The dose of CNP-22 was reported to be sufficient to enhance longitudinal growth of fetal tibias in organ culture experiment [39]. CNP-22 was purchased from Peptide Institute (4229-v, PEPTIDE INSTITUTE, Ibaraki, Japan). The culture duration was determined in accordance with our previous report including the organ culture experiment using tibias from fetal rats [35]. RNA extraction, reverse-transcription, and quantitative PCR analysis were performed as described above. Results were normalized using *Hprt1* and *Ppia* as reference genes.

For *in vitro* experiments, ATDC5 cells were plated at 1.0 × 10^5^ cells/well in 6-well tissue culture plates. ATDC5 cells were differentiated into proliferative chondrocytes by incubation with 10 μg/ml bovine insulin (No. 10516, SIGMA) for 14 days. Differentiated ATDC5 cells were incubated with vehicle or 10^−7^ M CNP-22 for 4 hours or 24 hours. RNA extraction, reverse-transcription, and quantitative PCR analysis were performed as described above. Because ATDC5 is a cell line from murine teratoma, primers were designed to detect murine *Nppc*, a coding gene for CNP. Results were normalized using murine hypoxanthine guanine phosphoribosyl transferase (*Hprt)* and murine peptidylprolyl isomerase A (*Ppia*) as reference genes.

The primers used in this analysis are listed in S1 Table. *Nppc* is a highly conserved gene and the sequences of primers for rat *Nppc* and murine *Nppc* were the same.

### Statistical analysis

Data are expressed as means ± SE in the text and depicted as dot and box plots with cross marks at means and bold lines at medians. Boxes represent interquartile ranges and whiskers represent minima and maxima with the exception of outliers (1.5 times out of interquartile range). Statistical analysis of the data was performed using either Student’s t-test or one-way factorial analysis of variance (ANOVA), followed by the Tukey–Kramer test as a post hoc test. The differences were considered significant when P values were less than 0.05.

## Results

### The effect of CNP on circulating NT-proCNP

**Fig 1:**
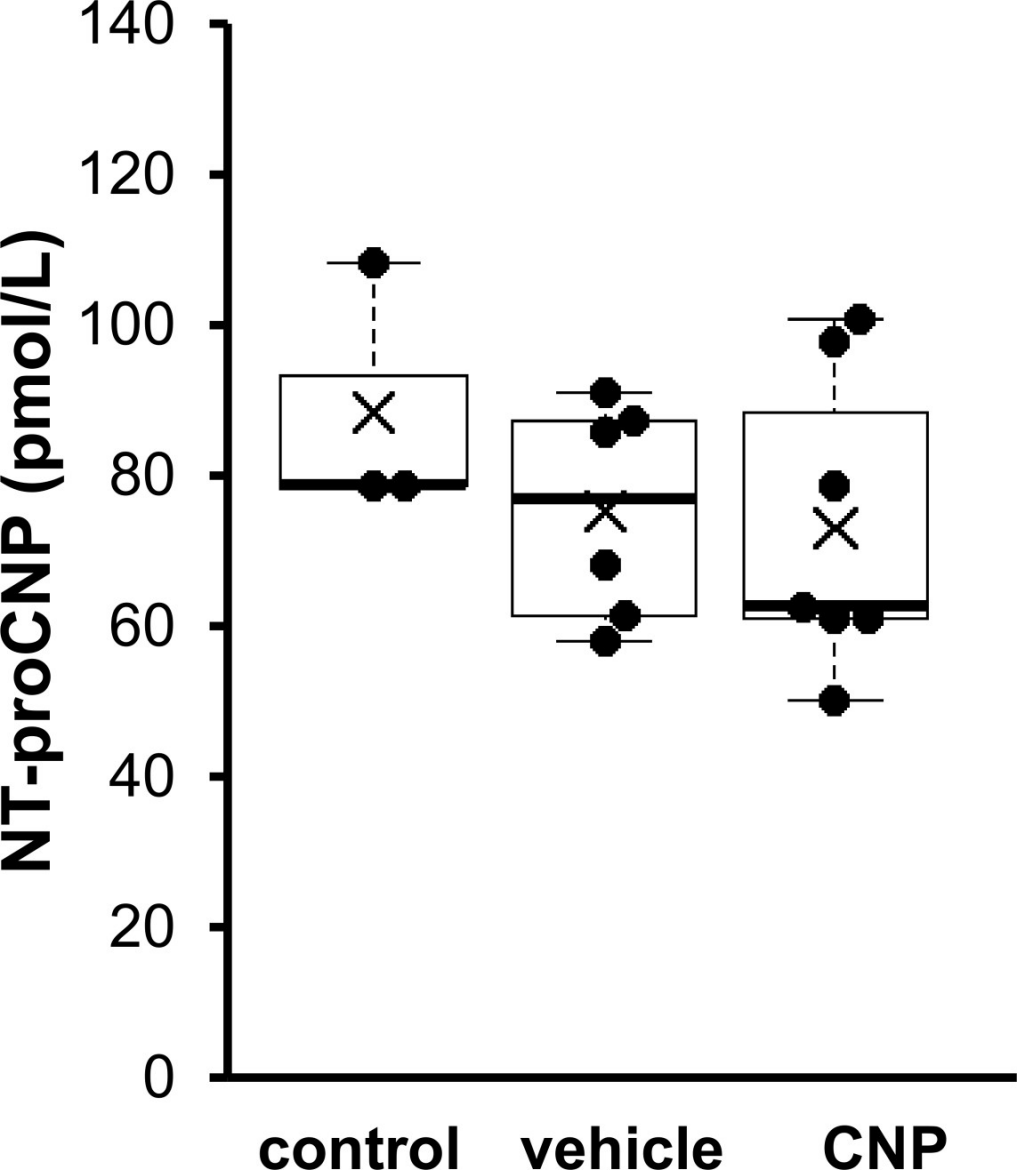
The effect of CNP administration on circulating NT-proCNP. Serum NT-proCNP levels after 3 days of vehicle or CNP treatment and control group. n = 3, 6 and 7, in control, vehicle-, and CNP-treated rats, respectively.

To explore whether CNP had a feedback effect or not, we investigated the serum NT-proCNP level as a marker of CNP production in wild-type rats under treatment with vehicle or CNP. We administered vehicle or 0.5 mg/kg/day of CNP for 3 days to 4-week-old male rats via osmotic pump. As depicted in Fig 1, we measured NT-proCNP levels and found that those in vehicle- and CNP-treated rats were comparable to control rats (88.81 ± 9.99 pmol/L, 75.47 ± 5.95 pmol/L, and 73.30 ± 7.04 pmol/L in control, vehicle-, and CNP-treated rats, respectively; n = 3, 6 and 7, in control, vehicle-, and CNP-treated rats, respectively). To exclude the cross-reaction between exogenous CNP and endogenous CNP products we measured NT-proCNP levels in CNP knockout rats treated with vehicle or CNP. Circulating NT-proCNP levels were below the limit of detection in both vehicle- and CNP-treated CNP knockout mice. These findings suggest that CNP is not regulated by positive or negative feedback mechanisms.

### The local effect of CNP on CNP production in CNP abundant tissues

**Fig 2:**
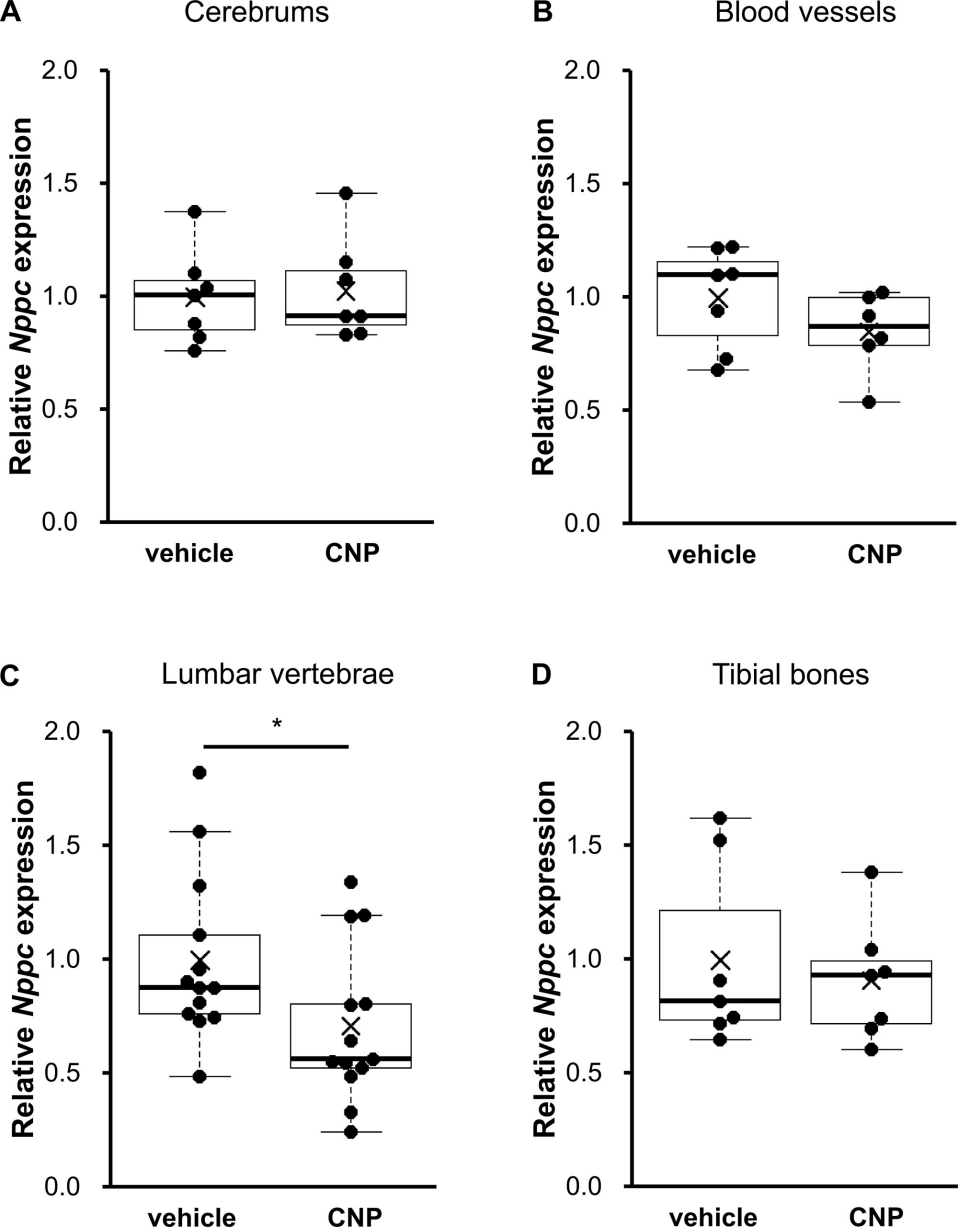
The effect of CNP administration on local *Nppc* expression. *Nppc* expression in (A) cerebrums, (B) blood vessels, (C) lumbar vertebrae, and (D) tibial diaphyses after 3 days of vehicle or CNP treatment were measured. The mRNA levels of *Nppc* were normalized by using rat (A) *Gusb*, (B) *Ywhaz*, and (C, D) *Hprt1* as reference genes, respectively. The data are represented as fold-change versus the values for vehicle-treated rats. (A, D) n = 7 for each of the vehicle- and CNP-treated rats. (B) n = 7 and 6, in vehicle-and CNP-treated rats, respectively. (C) n = 13 for each of the vehicle- and CNP-treated rats. *: P < 0.05.

Next we focused on local CNP production in wild-type rats and measured CNP expression in each tissue abundant in CNP, i.e., central nervous tissue, cardiovascular tissue, and skeletal tissue including cartilage. Fig 2A and 2B show that the mRNA expression of *Nppc*, a coding gene for CNP, is not changed by exogenous CNP in cerebrums (1.03 ± 0.08-fold in CNP-treated rats, P = 0.21, n = 7 for each group) or blood vessels (0.85 ± 0.07-fold in CNP-treated rats, P = 0.21, n = 7 and 6, in vehicle-and CNP-treated rats, respectively). As shown in Fig 2C, *Nppc* expression in lumbar vertebrae, including cartilaginous tissues, was significantly decreased by CNP administration (0.71 ± 0.10-fold in CNP-treated rats, P = 0.034, n = 13 for each group). For further investigation into skeletal tissues, we analyzed the change of *Nppc* expression in tibial diaphyses without growth plates; as depicted in Fig 2D, there was no significant change in *Nppc* expression (0.82 ± 0.11-fold in CNP-treated rats, P = 0.38, n = 7 for each group). These results suggest that *Nppc* expression in growth plates *in vivo* is reduced by systemic CNP administration. Data shown in Fig 2A–2D were normalized to one reference gene (*Gusb*, *Ywhaz*, *Hprt1*, and *Hprt1*, respectively) and the same results were obtained when another reference gene (*Ywhaz*, *Gapdh*, *Ppia*, and *Ppia*, respectively) was used (S1 Fig).

To analyze long term effect of CNP, CNP expression in lumbar vertebrae of 4-week-old mice and SAP-*Nppc*-Tg mice were also measured. *Nppc* expression in SAP-*Nppc*-Tg mice tended to be lower than that in wild-type mice but the CNP effect did not seem to be clearly enhanced by the long term overexposure (0.71 ± 0.11-fold in CNP-treated rats, P = 0.13, n = 7 for each group). Data was normalized to *Hprt* and the same result was obtained when *Ppia* was used as a reference gene (0.77 ± 0.08-fold in CNP-treated rats, P = 0.20, n = 7 for each group).

### The effect of CNP on female rats

**Fig 3:**
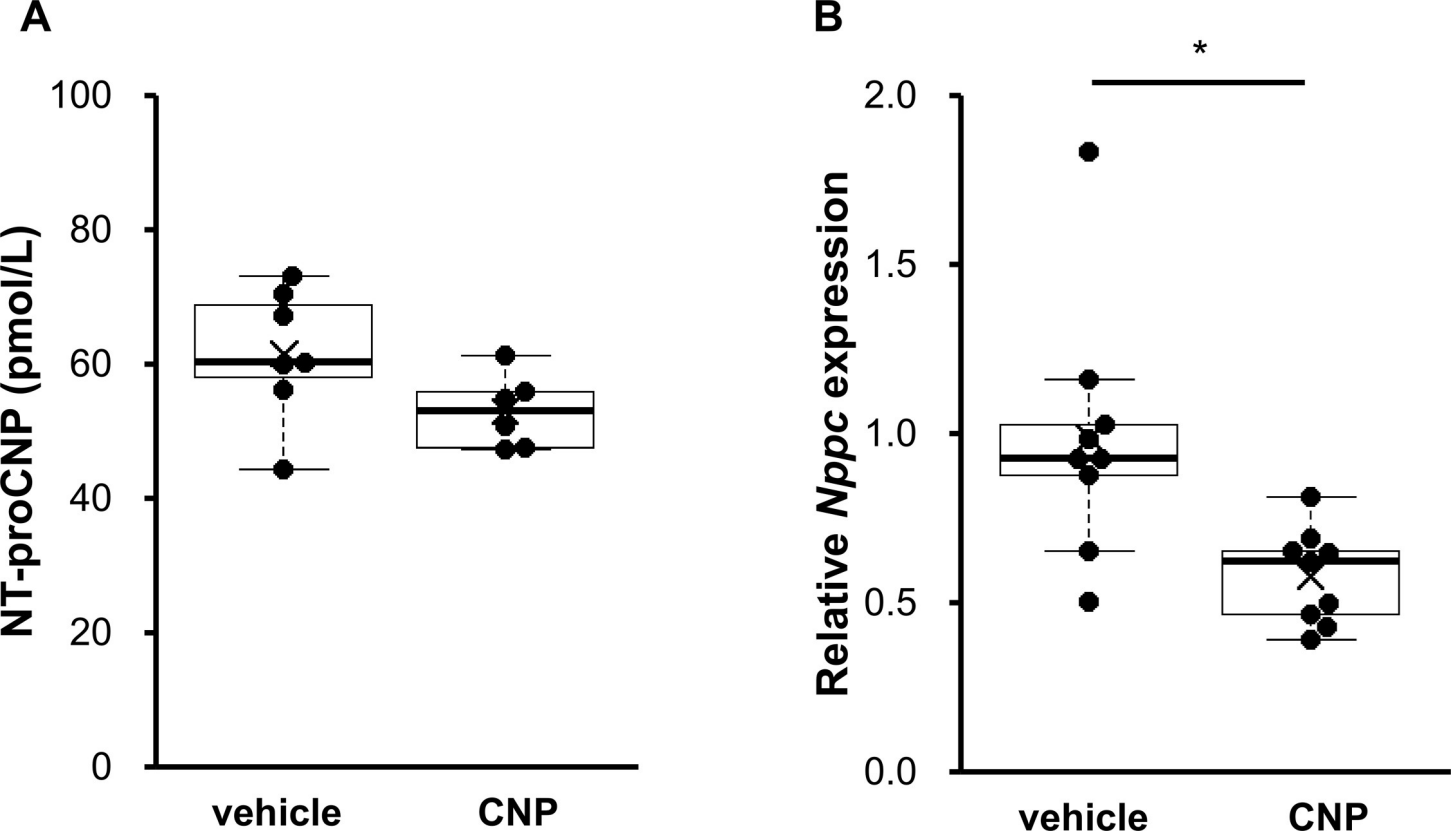
The effect of CNP administration on circulating NT-proCNP and local *Nppc* expression in female rats. (A) serum NT-proCNP levels and (B) *Nppc* expression in lumbar vertebrae after 3 days of vehicle or CNP treatment was measured in female rats. The mRNA levels of *Nppc* were normalized to rat *Hprt1* as a reference gene. The data are represented as fold-change versus the values for vehicle-treated rats. (A) n = 7 and 6, in vehicle-and CNP-treated rats, respectively. (B) n = 9 for each of the vehicle- and CNP-treated rats. *: P < 0.05.

Although CNP production in female rats might be variable during the estrus cycle, investigation of female rats would be useful for further understanding of CNP regulation. As we got important results in blood NT-proCNP levels and *Nppc* expression in lumbar vertebrae in male rats, we also measured them in female rats. As shown in Fig 3A, NT-proCNP levels in female rats were measured and similar results were obtained (61.55 ± 3.7 pmol/L vs. 52.81 ± 2.23 pmol/mg, P = 0.079, n = 7 and 6, in vehicle-and CNP-treated rats, respectively), although NT-proCNP levels in CNP-treated rats tended to slightly decrease. NT-proCNP levels in female rats treated with vehicle tend to be lower than those in male rats (88.81 ± 9.99 pmol/L vs. 61.55 ± 3.7 pmol/L, P = 0.065, n = 6 and 7, in male and female rats, respectively). As shown in Fig 3B, *Nppc* expression in female lumbar vertebrae also decreased by CNP administration (0.59 ± 0.05-fold in CNP-treated rats, P = 0.007, n = 9 for each group). Data was normalized to *Hprt1* and the same result was obtained when *Ppia* was used as a reference gene (S2 Fig).

### The effect of CNP on tibial growth plate

**Fig 4:**
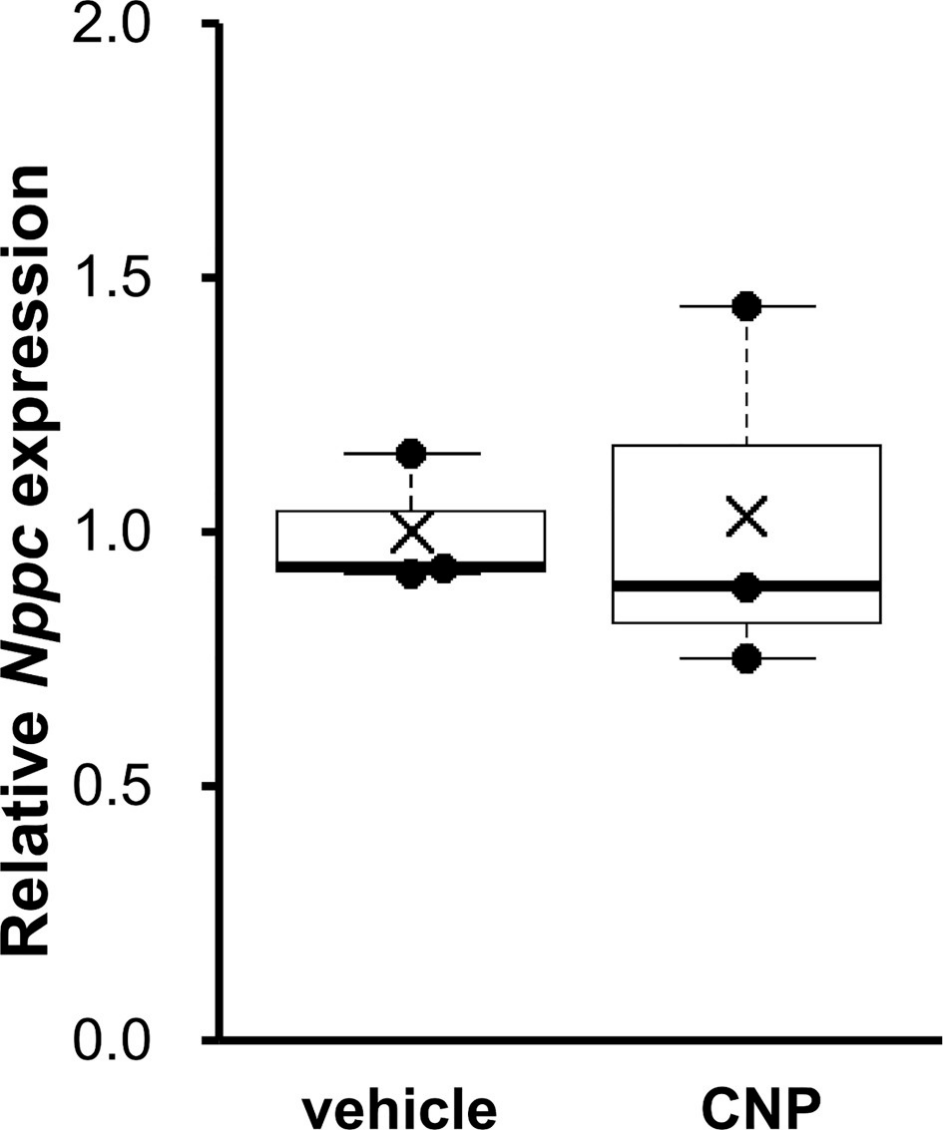
*Nppc* expression in organ culture experiment using tibias of fetal rats incubated with vehicle or CNP for 24 hours. The mRNA levels of *Nppc* were normalized to rat *Hprt1* as the reference gene. The data are represented as fold-change versus the values for vehicle-treated tibias; n = 3 for each of the vehicle- and CNP-treated tibias.

For further study on growth plates, we performed an *ex vivo* organ culture experiment by using appendicular growth plates isolated from fetal rats. As depicted in Fig 4, incubation for 24 hours with CNP did not significantly change *Nppc* expression (1.03 ± 0.21-fold in CNP-treated limbs, P = 0.90, n = 3 for each group). Data shown in Fig 4 were normalized to *Hprt1* and the same result was obtained when *Ppia* was used as a reference gene (S3 Fig).

### CNP regulation in differentiated ATDC5 cells

**Fig 5:**
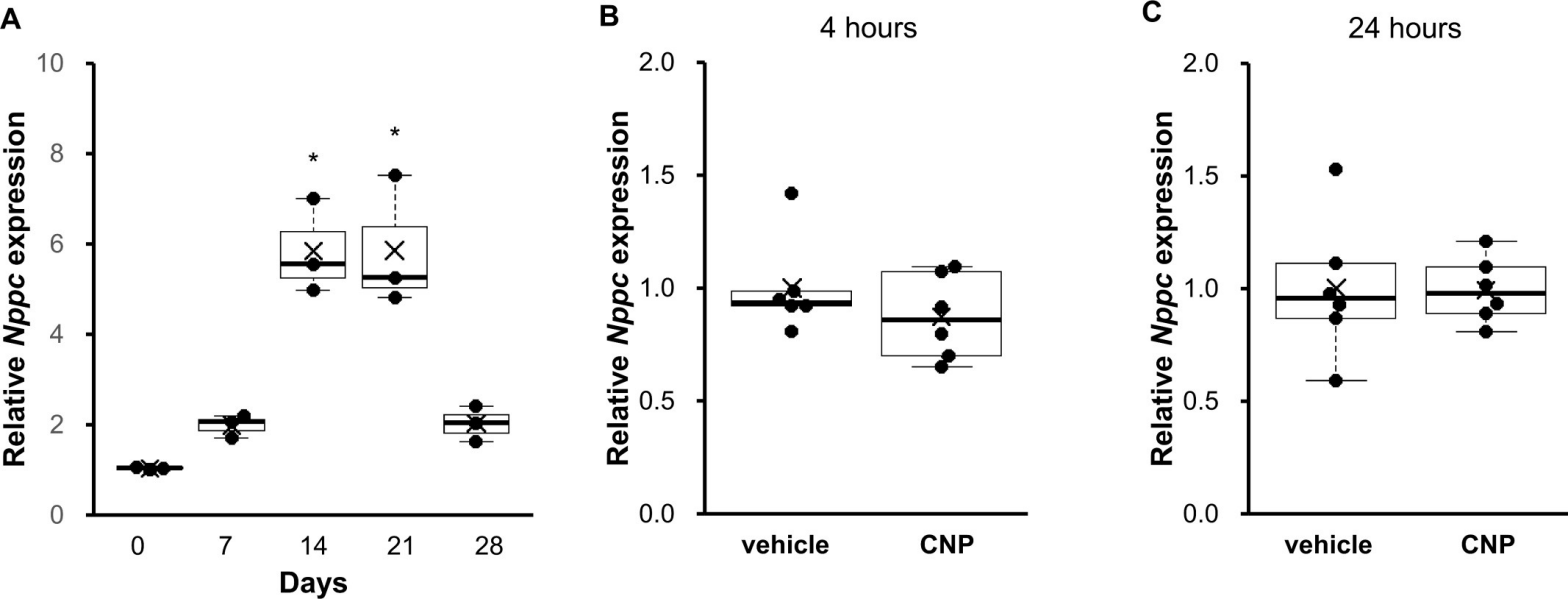
*In vitro* experiments using the ATDC5 cell line. (A) The change in *Nppc* mRNA levels of ATDC5 cells differentiated by incubation with bovine insulin. (B, C) The effect of CNP on *Nppc* mRNA expression in differentiated ATDC5 cells incubated with vehicle or CNP for 4 hours (B) and for 24 hours (C). The mRNA levels were normalized to murine *Hprt* as the reference gene. The data are represented as fold-change versus the values for cells at day 0 (A) and for vehicle-treated cells (B, C). (A) n = 3 for each time point. *: P < 0.05 vs. day 0. (B, C) n = 6, each, in the vehicle- and CNP-treated cells.

Finally, we performed experiments using the chondrogenic cell line ATDC5, which mimics a differentiating growth plate chondrocyte, in order to evaluate the CNP effect on a single layer of chondrocytes. We confirmed that incubation with insulin for 14 days sufficiently differentiated ATDC5 into CNP-producing chondrocytes (Fig 5A). Incubation with CNP for 30 minutes successfully elevated cGMP levels. cGMP concentration in culture medium was almost undetectable (0.01 ± 0.03 pmol/ml) when incubated with vehicle and markedly elevated (85.36 ± 7.61 pmol/ml) when incubated with CNP (P = 0.0004, n = 3 for each group). However, as shown in Fig 5B, incubation with CNP for 4 hours did not increase *Nppc* expression in differentiated chondrocytes (0.90 ± 0.05-fold in CNP-treated cells, P = 0.29, n = 6 for each group). Fig 5C shows the *Nppc* expression after incubation with vehicle or CNP for 24 hours. CNP did not change *Nppc* expression in differentiated chondrocytes even after incubation for 24 hours (0.99 ± 0.06-fold in CNP-treated cells, P = 0.95, n = 6 for each group). Data shown in Fig 4 were normalized to *Hprt* and the same result was obtained when *Ppia* was used as a reference gene (S4 Fig).

### The effect of CNP on local factors involved in the activity of CNP

**Fig 6:**
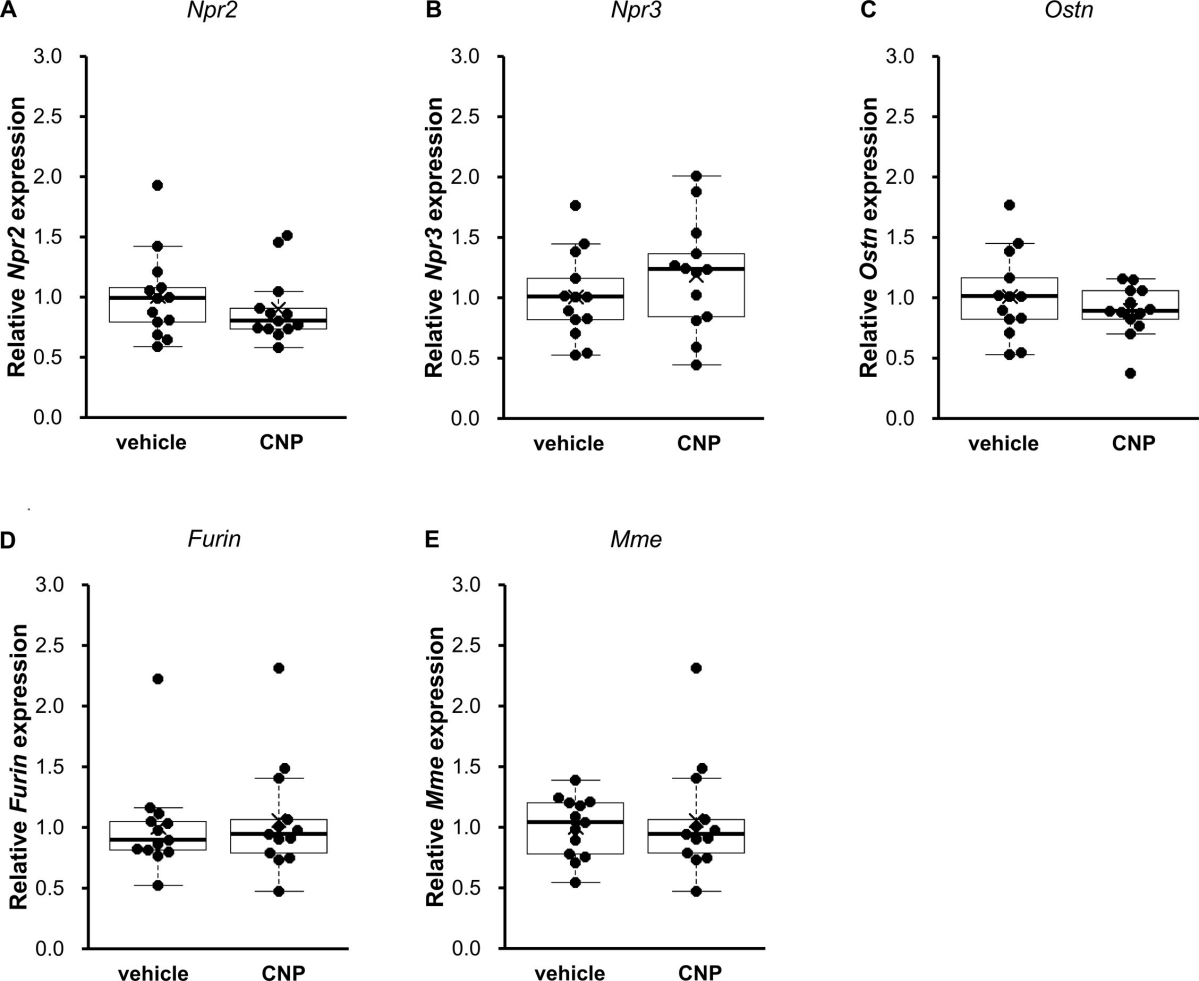
The effect of CNP administration on local factors regulating CNP effect. The expression of (A) *Npr2*, (B) *Npr3*, (C) *Ostn*, (D) *Furin*, and (E) *Mme* in lumbar vertebrae after 3 days of vehicle or CNP treatment were measured. The mRNA levels were normalized to rat *Hprt1* as a reference gene. The data are represented as fold-change versus the values for vehicle-treated rats. n = 13 for each of the vehicle- and CNP-treated rats.

Next we focused on factors which affects the action, production, and degradation of CNP. As these factors, expression of NPR-B, natriuretic peptide receptor C (NPR-C), osteocrin (OSTN), furin, neutral endopeptidase (NEP) were measured. Their coding genes are *Npr2*, *Npr3*, *Ostn*, *Furin*, and *Mme*, respectively. As shown in Fig 6, CNP administration did not changed the mRNA expression of *Npr2* (0.91 ± 0.07-fold in CNP-treated rats, P = 0.44, n = 13 for each group), *Npr3* (1.15 ± 0.13-fold in CNP-treated rats, P = 0.43, n = 13 for each group), *Ostn* (0.90 ± 0.08-fold in CNP-treated rats, P = 0.33, n = 13 for each group), *Furin* (1.07 ± 0.10-fold in CNP-treated rats, P = 0.49, n = 13 for each group), or *Mme* (1.00 ± 0.07-fold in CNP-treated rats, P = 0.97, n = 13 for each group) in lumbar vertebrae. Data were normalized to *Hprt1* and the same results were obtained when *Ppia* was used as a reference gene (S5 Fig).

## Discussion

In the present study, we investigated the regulation of CNP levels in rodent models. To begin, we studied whether autoregulation of CNP exists in biological systems, i.e., endocrinological feedback regulation. Past studies on circulating CNP levels include clinical studies [40–44] and *in vivo* studies using animals [30, 45–48]. Although there are a few clues about CNP feedback in previous clinical reports, it is controversial. Here, we used a rat injection model. As for the setting of CNP stimulation, we adopted continuous subcutaneous injection of CNP and decided to estimate the CNP levels three days after the start of administration. We administered exogenous CNP-53 at the same dose that previously restored the dwarfism of CNP knockout rats [35] and found that sufficient exogenous CNP injection had little if any effect on circulating NT-proCNP levels. The result in male rats is not supportive for the existence of feedback regulation of CNP, but local production and content of CNP would be important for understanding CNP action and regulation [32]. Indeed, CNP is produced in various tissues and not all circulating CNP is derived from the growth plate [49]. Thus circulating CNP levels would be reflected by the sum of secreted CNP from these tissues. Furthermore, It is important to keep in mind that differential responses of CNP signaling pathways likely occur across tissues, resulting in different regulatory effects by CNP; for example, CNP suppresses ERK phosphorylation in chondrocytes [13, 31], whereas it promotes ERK phosphorylation in pituitary cells [50]. Therefore, if CNP is positively regulated in some tissues and negatively regulated in other tissues, the sum of circulating NT-proCNP might not be changed.

Next we focused on local CNP production by elevating the levels of CNP and measured *Nppc* mRNA in each tissue in wild-type rats. We evaluated local CNP production in each tissue known to be abundant in CNP, for example, nervous system tissue, blood vessels, and growth plate cartilage, and found that *Nppc* expression was decreased only in growth plate cartilage. We used lumbar vertebrae as an approximation for growth plate tissue as it was difficult to obtain pure growth plate tissue *in vivo*. However, lumbar vertebrae consist of tissues other than the growth plate such as bone and bone marrow, and it is possible that a change in *Nppc* expression in lumbar vertebrae is due to the change in these tissues other than growth plate tissue. Therefore, we performed additional experiments using tibial diaphyses, which is bone tissue without a growth plate, and found *Nppc* expression was not changed in bone and bone marrow. These findings support the hypothesis that exogenous CNP decreases CNP expression only in growth plate cartilage.

In these experiments, both direct and indirect effect of CNP administration is observed. For further study in pure growth plate tissue as the main target site of the CNP/NPR-B signaling system, we performed an *ex vivo* study using fetal murine tibias to analyze direct effect of CNP on growth plate cartilage and showed that CNP production in the growth plate in explanted tibias are not changed by CNP treatment. However, the organ culture experiments include whole growth plates and might be inappropriate for the investigation into the direct effect of CNP on the CNP abundant layer of the growth plate. As an *in vitro* study, we investigated the direct effects of exogenous CNP on CNP expression in moderately differentiated ATDC5 cells, mimicking growth plate chondrocytes in the proliferative and prehypertrophic layers. Although *in vitro* studies on CNP regulation to date have been performed by using a pituitary cell line [33, 51] and granulosa cells [52], investigation into CNP regulation in cartilaginous cells is essential for the understanding of the growth effect of CNP. We examined two different treatment periods, 4 hours and 24 hours, targeting the first transcription and secondary or later transcriptional effect, respectively. We failed to find significant alteration of CNP expression by CNP.

CNP is produced by cleavage of proCNP and furin is an essential enzyme for the processing of proCNP. [10]. Produced CNP binds to NPR-B and CNP/NPR-B signaling is activated through cGMP production. This signaling system is affected by NPR-C, a clearance receptor of CNP, and OSTN, a specific ligand of NPR-C. CNP coupled with NPR-C is degraded and OSTN increases CNP activity by acting as an intrinsic ligand of NPR-C [53]. NEP is also related to rapid degradation of CNP [54]. For further understanding of the regulation of the CNP/NPR-B signaling system, we performed investigation on the local expressions of these factors involved in the regulation of CNP production, action, and degradation, which resulted no change in any of *Npr2*, *Npr3*, *Ostn*, *Furin*, and *Mme*. Intracellular cGMP produced by CNP/NPR-B signaling is degraded by phosphodiesterase (PDE) [55]. Thus changes in PDE would be alter CNP/NPR2 activity. In ATDC5 cells, when *Nppc* expression is raised through differentiation, cGMP hydrolytic activities of PDE1 and PDE5 are enhanced [56] and CNP is reported to enhance PDE2 which degrades cGMP and suppress PDE3 which degrades cAMP mainly [57]. Alteration of PDE activity due to CNP might be related to regulation of CNP/NPR-B activity.

Because CNP is actually produced in various tissues, *Nppc* alteration only in growth plates might not clearly change blood NT-proCNP level. It is reported that about 40% of circulating CNP is produced in endothelial cells [49], but the value of 40% is the result at 16 weeks of age when growth plates would be thinned. In younger rats whose growth plates are thicker, *Nppc* expression in growth plates would have more impact on the circulating NT-proCNP. Actually in humans, NT-proCNP levels in children are about 2–3 times higher than those in adults [58, 59]. Thus our experiments using 4-week-old rats are likely to emphasize the effect of local *Nppc* change on circulating NT-proCNP. Nevertheless, decreased *Nppc* expression in lumbar vertebrae did not clearly reduce circulating NT-proCNP levels in our experiments. Although growth plates might be the major source of circulating NT-proCNP in young rats, 30–40% decrease in cartilaginous *Nppc* expression might not be sufficient to significantly change circulating NT-proCNP levels because of the NT-proCNP from other tissues. Thus it might be difficult to decrease blood NT-proCNP level by enhancing growth plate CNP/NPR-B activity and consequent negative feedback on CNP gene expression in growth plates.

As for the effect of elevated circulating CNP levels in genetic disorders of growth in humans, the report on the loss-of-function mutation in *NPR3* is important. Deterioration of NPR3 causes the elevation of circulating CNP levels via the reduction of CNP degradation but it is not expected to affect NT-proCNP metabolism. Blood CNP and NT-proCNP levels are reported in two pediatric patients: CNP levels are elevated (1.6 SDS and 6.4 SDS, respectively) and blood NT-proCNP levels are slightly decreased (-0.7 SDS and -0.05 SDS, respectively) [60]. Drastic enhancement of CNP/NPR-B activity reduces NT-proCNP more clearly. Some patients with gain-of-function mutations of *NPR2* are reported to have decreased NT-proCNP levels [23, 26] but other patients are reported to have normal NT-proCNP levels [23, 27]. In reference 23, a child patient has lower NT-proCNP level and adult patients have normal NT-proCNP levels. The data support our results that CNP suppresses CNP production mainly in growth plate. However, the data should be interpreted carefully because of the lack of age-matched reference range [23].

We found slight decrease in blood NT-proCNP level due to CNP administration to female mice. The change in female mice might be due to viability related to estrus cycle, or difference in susceptibility to CNP. Blood NT-proCNP levels in female rats tend to be lower than those in male rats. If endogenous CNP levels in female rats are lower than those of male rats, exogenous CNP administration might be more effective in female rats. While further studies are needed on sex differences in CNP production and efficacy, the greater reduction of *Nppc* expression in growth plate of females (which would reflect activation of CNP/NPR-B by exogenous CNP) may connect with the reduction of circulating NT-proCNP levels. From the clinical and experimental data raised above, strong and sufficient activation of CNP/NPR-B would be necessary to reduce circulating NT-proCNP levels significantly.

As for the suppression of CNP/NPR-B signaling in genetic disorders of growth, drastic change in CNP/NPR-B signaling, for example, ablation of NPR-B, and subsequent critical change in *Nppc* expression in growth plate could be expected to change the circulating NT-proCNP level. NT-proCNP is increased in patients with biallelic loss-of-function mutations in *NPR2* [16, 21] while not increased in patients with monoallelic loss-of-function mutations in *NPR2* [16]. However, it should be noted that SDS of plasma NT-proCNP in patients with monoallelic loss-of-function mutations in *NPR2* have not been specifically addressed in children. As another condition with suppressed NPR-B activity, achondroplasia have been reported. Achondroplasia is a genetic disease which causes short stature due to a gain-of-function mutation in fibroblast growth factor receptor 3 (FGFR3). A recent study indicates that FGFR3 suppresses NRP-B activity through dephosphorylation of the juxtamembrane domain of NPR-B [61] and the impairment of NPR-B activity is related to the impaired skeletal growth shown in achondroplasia [62]. On the other hand circulating NT-proCNP levels are elevated in patients with achondroplasia [44], which suggests that FGFR-3 induced suppression of CNP/NPR-B signaling would increase *Nppc* expression.

In the present study, *Nppc* expression in growth plate cartilage was found to be decreased by CNP treatment through experiments in vivo but not decreased through experiments ex vivo and in vitro. These findings suggest that CNP is regulated by feedback system although surrounding tissues are required for the feedback regulation. For example, Indian hedgehog and parathyroid hormone-related protein (PTHrp), involved in hypertrophic differentiation of chondrocytes, form a negative feedback loop and PTHrp-positive chondrocytes in growth plate are reported to migrate to the bone tissue outside the growth plate [63]. As for the feedback regulation of CNP, cells outside the growth plate might secrete some factor that suppresses CNP production. The mechanism of regulating CNP is not well studied and this is an important theme in the future study. The result that *Nppc* expressions were not changed in tissues other than growth plate cartilage suggests the main site of CNP function would be cartilage tissue. Endothelial cells are abundant in CNP and its effect on endothelial cells are reported to be exerted through NPR-C instead of NPR-B [64]. Feedback regulation of CNP would require activation of NPR-B and its downstream. The exact pathway of feedback is unclear and further studies are needed.

In conclusion, we showed that exogenous CNP decreased endogenous CNP production in growth plate cartilage through *in vivo* experiments. The regulatory effect seems to be indirect because exogenous CNP did not change CNP production through *ex vivo and in vitro* experiments. Regulation of the CNP effect is enigmatic because CNP transcription is not well studied and the CNP effect would be controlled not only by CNP production but also by CNP clearance. More studies are required to further elucidate the regulatory system of CNP production and function.

## Supporting information

S1 Fig The effect of CNP administration on local *Nppc* expression normalized by another reference gene. *Nppc* expression in (A) cerebrums, (B) blood vessels, (C) lumbar vertebrae, and (D) tibial bones after 3 days of vehicle or CNP treatment were measured. The mRNA levels of *Nppc* were normalized to rat (A) *Ywhaz*, (B) *Gapdh*, and (C, D) *Ppia* as the reference genes, respectively. The data are represented as fold-change versus the values for vehicle-treated rats. (A, D) n = 7 for each of the vehicle- and CNP-treated rats. (B) n = 7 and 6, in vehicle-and CNP-treated rats, respectively. (C) n = 13 for each of the vehicle- and CNP-treated rats. *: P < 0.05.
(TIF)

S2 Fig The effect of CNP administration on local *Nppc* expression in female rats normalized by another reference gene. *Nppc* expression in lumbar vertebrae after 3 days of vehicle or CNP treatment was measured in female rats. The mRNA levels of *Nppc* were normalized to rat *Ppia* as a reference gene. The data are represented as fold-change versus the values for vehicle-treated rats. n = 9 for each of the vehicle- and CNP-treated rats.
(TIF)

S3 Fig *Nppc* expression in the organ culture experiment using tibias of fetal rats incubated with vehicle or CNP for 24 hours, normalized to another reference gene. The mRNA levels of *Nppc* were normalized to rat *Ppia* as the reference gene. The data are represented as fold-change versus the values for vehicle-treated tibias; n = 3 in each of the vehicle- and CNP-treated tibias.
(TIF)

S4 Fig *In vitro* experiments of the ATDC5 cell line using another reference gene. (A) The change of *Nppc* mRNA levels of ATDC5 cells differentiated by incubation with bovine insulin. (B, C) The effect of CNP on *Nppc* mRNA expression in differentiated ATDC5 cells incubated with vehicle or CNP for 4 hours (B) and 24 hours (C). The mRNA levels were normalized to murine *Ppia* as the reference gene. The data are represented as fold-change versus the values for cells at day 0 (A) and for vehicle-treated cells (B, C). (A) n = 3 for each time point. *: P < 0.05 vs. day 0. (B, C) n = 6, in each of the vehicle- and CNP-treated cells.
(TIF)

S5 Fig The effect of CNP administration on local factors regulating CNP effect normalized by another reference gene. The expression of (A) *Npr2*, (B) *Npr3*, (C) *Ostn*, (D) *Furin*, and (E) *Mme* in lumbar vertebrae after 3 days of vehicle or CNP treatment were measured. The mRNA levels were normalized to rat *Ppia* as a reference gene. The data are represented as fold-change versus the values for vehicle-treated rats. n = 13 for each of the vehicle- and CNP-treated rats.
(TIF)

S1 Table Primer sequences used for quantitative RT-PCR analysis. *Nppc* is a highly conserved gene and the primer sequences of rat *Nppc* and murine *Nppc* were the same.
(TIF)
