# Supplementary figures and images for: Is C-type natriuretic peptide regulated by a feedback loop? A study on systemic and local autoregulatory effect
Source: PLoS One. 2020 Oct 1;15(10):e0240023. doi: 10.1371/journal.pone.0240023 (PMC7529242; doi:10.1371/journal.pone.0240023)

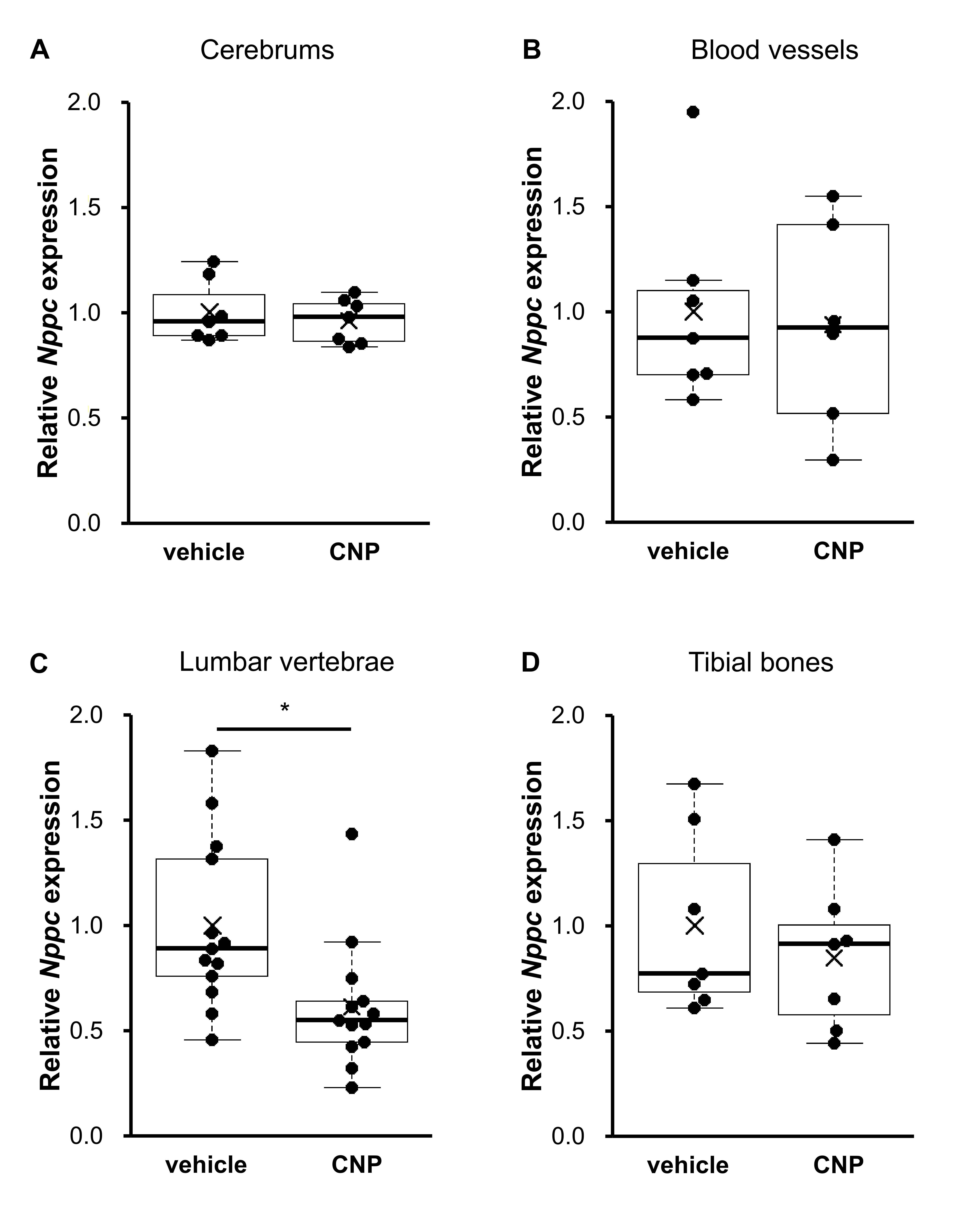

Supplement: S1 Fig — Nppc expression in (A) cerebrums, (B) blood vessels, (C) lumbar vertebrae, and (D) tibial bones after 3 days of vehicle or CNP treatment were measured. The mRNA levels of Nppc were normalized to rat (A) Ywhaz, (B) Gapdh, and (C, D) Ppia as the reference genes, respectively. The data are represented as fold-change versus the values for vehicle-treated rats. (A, D) n = 7 for each of the vehicle- and CNP-treated rats. (B) n = 7 and 6, in vehicle-and CNP-treated rats, respectively. (C) n = 13 for each of the vehicle- and CNP-treated rats. *: P < 0.05. (TIF) [file pone.0240023.s001.tif]

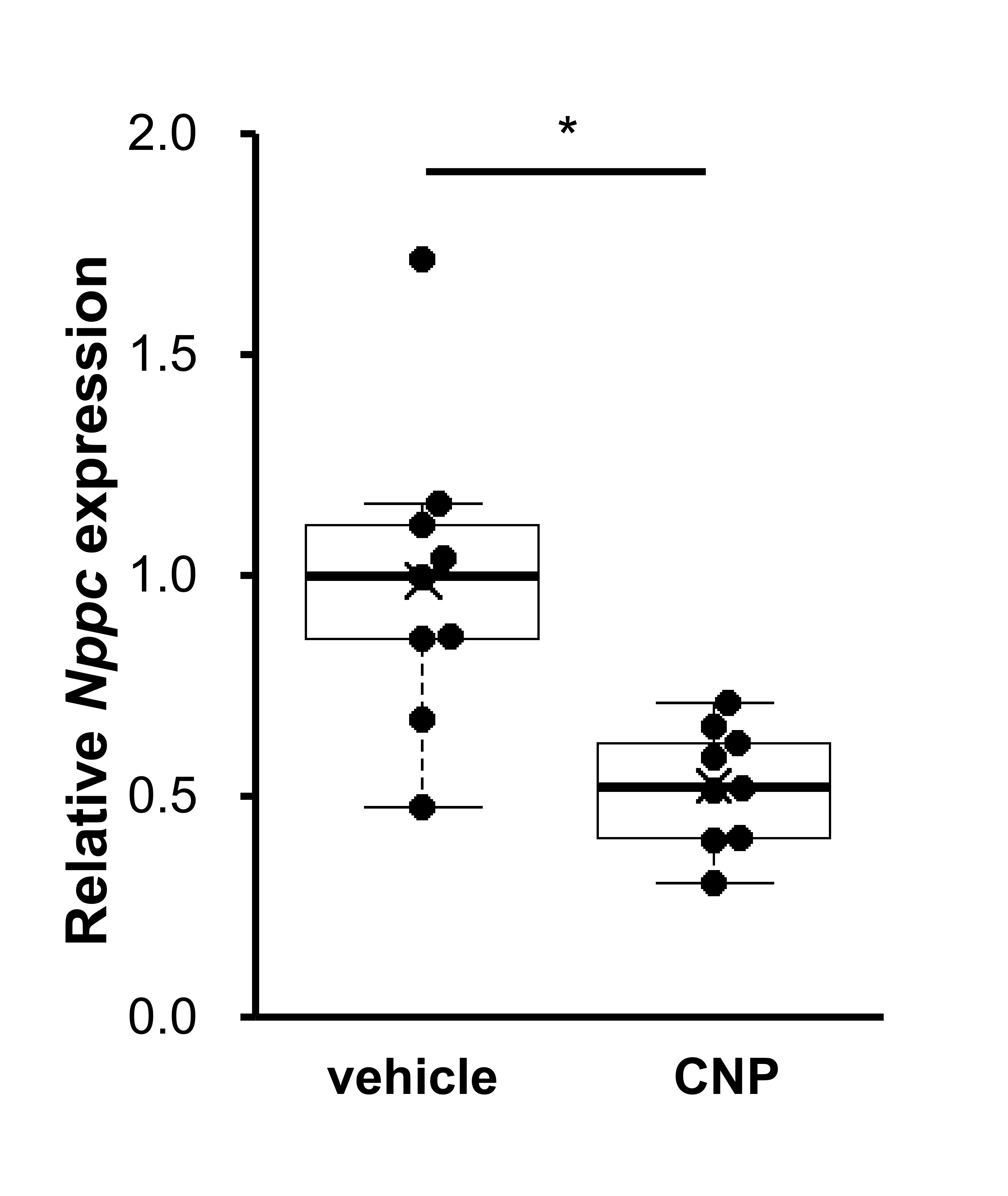

Supplement: S2 Fig — Nppc expression in lumbar vertebrae after 3 days of vehicle or CNP treatment was measured in female rats. The mRNA levels of Nppc were normalized to rat Ppia as a reference gene. The data are represented as fold-change versus the values for vehicle-treated rats. n = 9 for each of the vehicle- and CNP-treated rats. (TIF) [file pone.0240023.s002.tif]

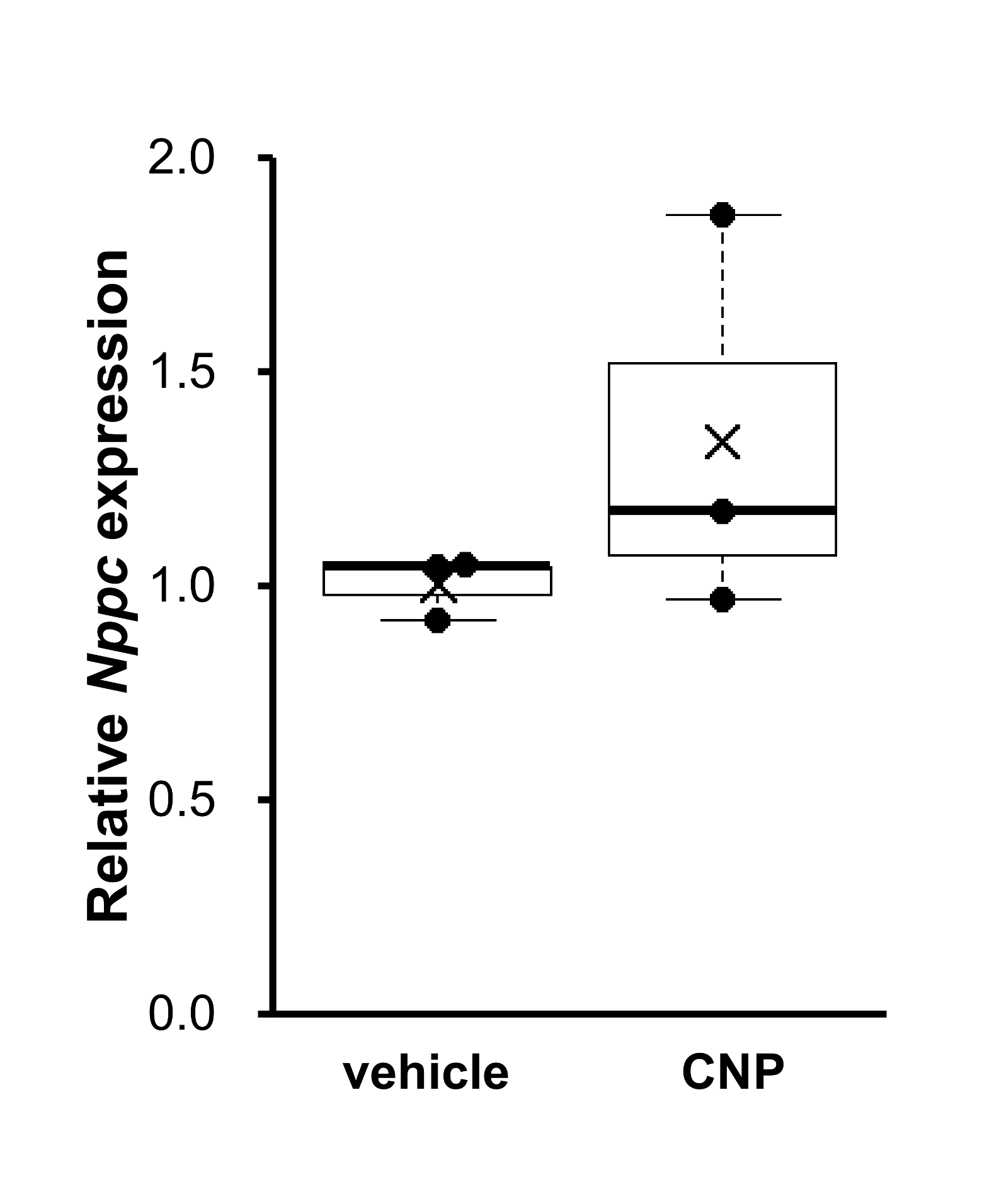

Supplement: S3 Fig — The mRNA levels of Nppc were normalized to rat Ppia as the reference gene. The data are represented as fold-change versus the values for vehicle-treated tibias; n = 3 in each of the vehicle- and CNP-treated tibias. (TIF) [file pone.0240023.s003.tif]

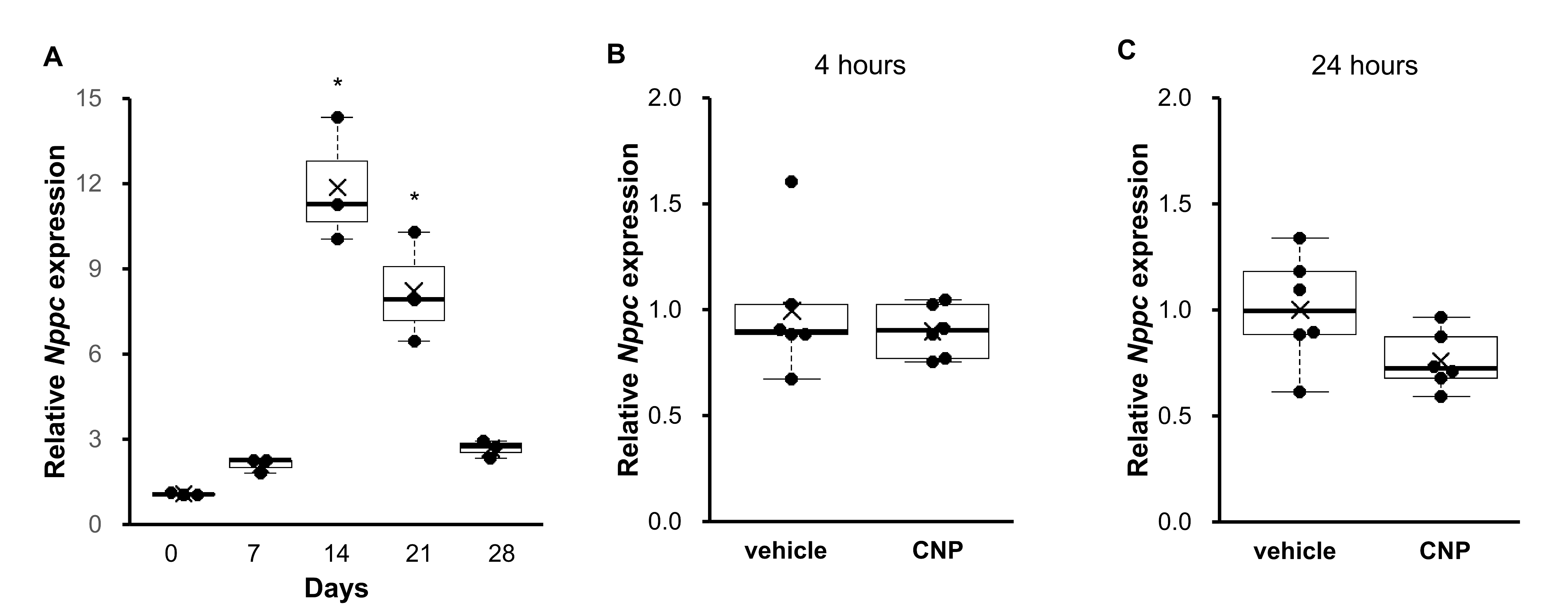

Supplement: S4 Fig — (A) The change of Nppc mRNA levels of ATDC5 cells differentiated by incubation with bovine insulin. (B, C) The effect of CNP on Nppc mRNA expression in differentiated ATDC5 cells incubated with vehicle or CNP for 4 hours (B) and 24 hours (C). The mRNA levels were normalized to murine Ppia as the reference gene. The data are represented as fold-change versus the values for cells at day 0 (A) and for vehicle-treated cells (B, C). (A) n = 3 for each time point. *: P < 0.05 vs. day 0. (B, C) n = 6, in each of the vehicle- and CNP-treated cells. (TIF) [file pone.0240023.s004.tif]

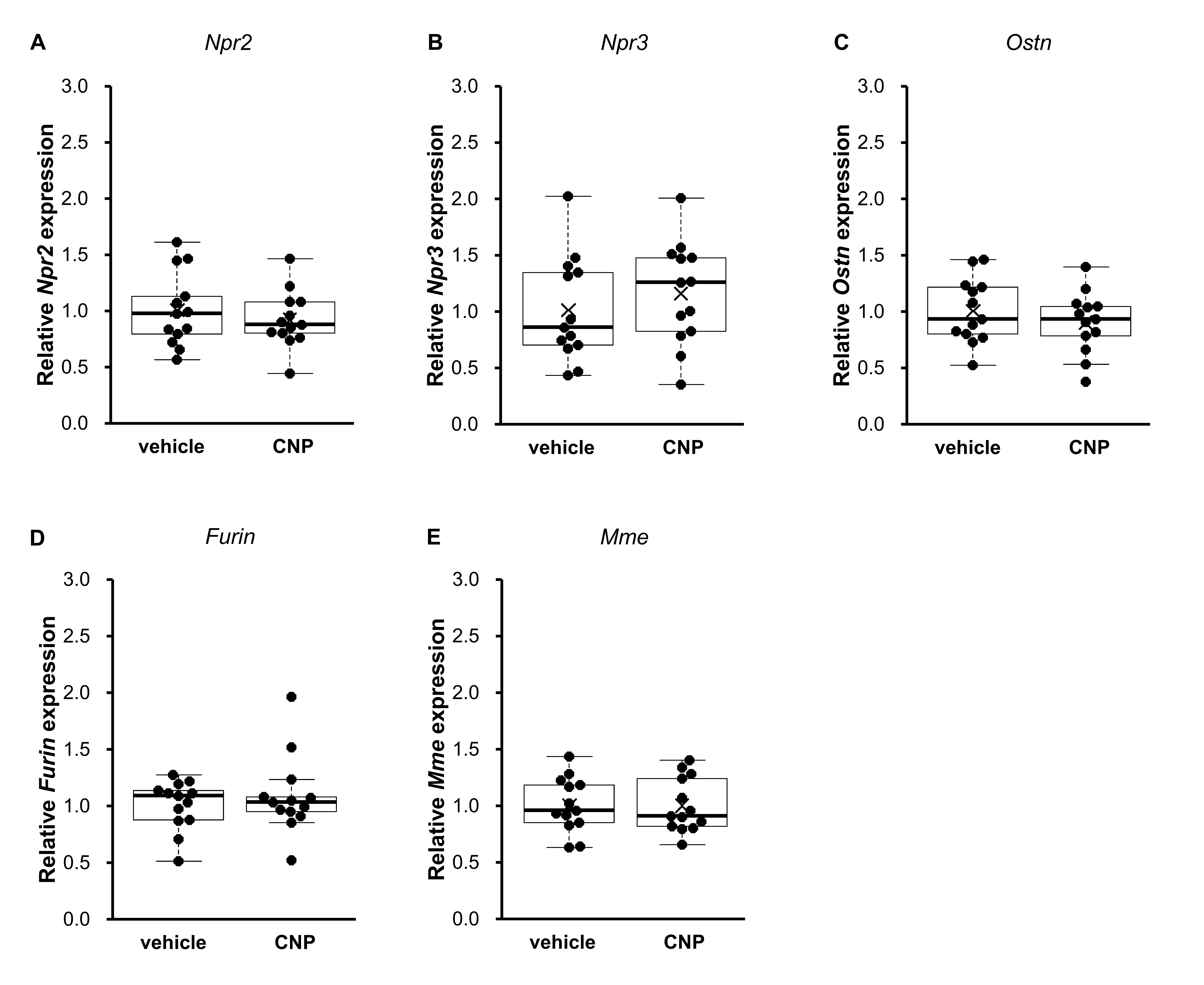

Supplement: S5 Fig — The expression of (A) Npr2, (B) Npr3, (C) Ostn, (D) Furin, and (E) Mme in lumbar vertebrae after 3 days of vehicle or CNP treatment were measured. The mRNA levels were normalized to rat Ppia as a reference gene. The data are represented as fold-change versus the values for vehicle-treated rats. n = 13 for each of the vehicle- and CNP-treated rats. (TIF) [file pone.0240023.s005.tif]

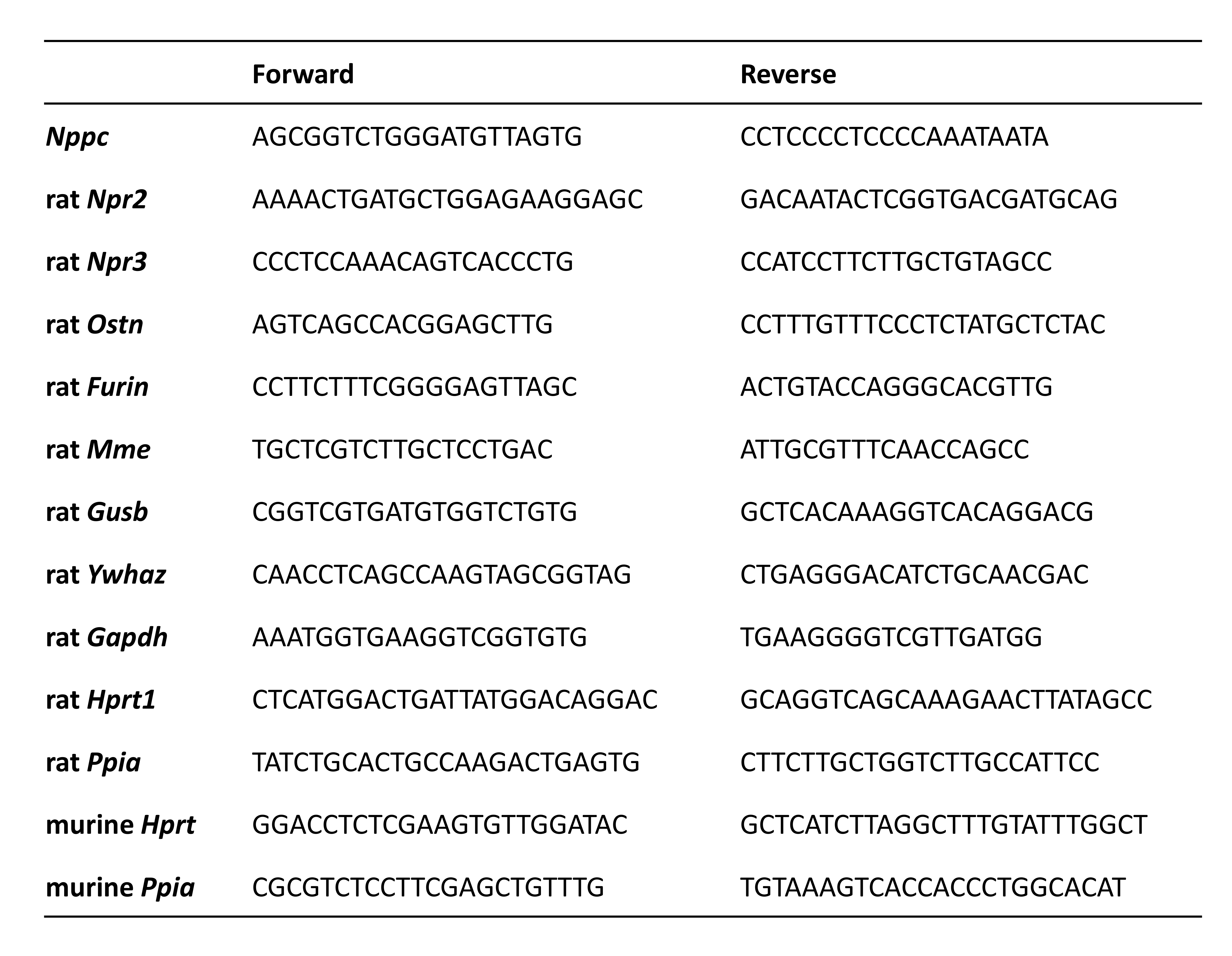

Supplement: S1 Table — Nppc is a highly conserved gene and the primer sequences of rat Nppc and murine Nppc were the same. (TIF) [file pone.0240023.s006.tif]
